# Comparison Between Clinical Outcome of Intralesional Human Placental Extract Alone and Topical Application of Placental Extract Gel After Fibrotomy in Oral Submucous Fibrosis

**DOI:** 10.7759/cureus.40105

**Published:** 2023-06-07

**Authors:** Lalrin Zuali, S. M. Azeem Mohiyuddin, Sagayaraj A, Kouser Mohammadi, Indranil Paul

**Affiliations:** 1 Otorhinolaryngology and Head and Neck Surgery, Sri Devaraj Urs Medical College, Kolar, IND

**Keywords:** placental extract gel, fibrotomy, intra-lesional placental extract injection, trismus, oral submucous fibrosis

## Abstract

Background: Oral submucous fibrosis (OSMF) is a premalignant condition prevalent in our country. Juxtaepithelial inflammation with progressive hyalinization of the lamina propria results in stiffness and fibrosis of the oral mucosa, characterised by trismus, ankyloglossia, and a burning sensation. Various methods of treatment have been tried in these cases, which include placental extract injections and the cutting of fibrous bands. In this study, we aim to compare the outcome of intra-lesional placental extract injection with fibrotomy and placental extract gel application in OSMF.

Methodology: This prospective interventional study included 58 patients clinically diagnosed with OSMF grades II and III at a rural tertiary care hospital between January 2021 and August 2022. The patients were randomised into two groups: group I received 1 ml of intra-lesional human placental extract injection in the submucosal plane of the buccal mucosa and retro-molar trigone (RMT) once a week for five consecutive weeks, and group II was subjected to a transverse division of fibrotic bands in the submucosal plane under general anaesthesia. The surgical wound was left open, and swabs soaked in human-purified placental extract gel were placed in the wound for two hours twice daily until the surgical wound was epithelialized and healed. The patients in both groups I and II were advised to do jaw opening exercises, and weekly follow-up was done. Findings with regard to maximum mouth opening, colour of mucosa, and burning sensation in the oral cavity based on a Likert scale were documented. At the end of five months, the pre-treatment and post-treatment results documented were compared.

Results: All patients were between 20 and 60 years of age and were addicted to chewing areca nuts with tobacco. Bilateral involvement was present in all patients, with extension into the RMT and soft palate seen in 31%. Improvement in mouth opening was between 4 mm and 6 mm in group II, and relief of burning sensation and mucosal colour was better in group I.

Conclusion: Intra-lesional placental extract injections help in the improvement of the mucosa and relief from the burning sensation. Fibrotomy with placental extract gel application is better at relieving trismus in OSMF. Aggressive mouth-opening exercises may improve mouth opening following the above procedures.

## Introduction

Oral submucous fibrosis (OSMF) is a premalignant condition prevalent in India, South Africa, and Middle Eastern countries. In this condition, juxtaepithelial inflammation with progressive hyalinization of the lamina propria results in stiffness and fibrosis in the mucosa of the oral cavity, thereby leading to trismus and ankyloglossia [1]. It also causes a burning sensation in the oral cavity. Major risk factors and carcinogens include addiction to betel nut or areca nut chewing with slaked lime, smoking and tobacco chewing, high copper content in food, and spicy food having a high content of capsaicin [2].

In India, the prevalence of oral cancer has been increasing, and it is the most common malignancy in our region. The prevalence of OSMF in India was estimated to range from 0.2% to 2.3% in males and 1.2% to 2.6% in females, and it is found to be more common in the southern part of India [2]. The main cause of this high prevalence in our region is addiction to chewable tobacco, areca nuts, and betel leaves. Malignant transformation occurs in 7-30% of OSMF cases [3]. It causes significant morbidity due to a burning sensation in the oral cavity, trismus, and ankyloglossia.

Various methods of treatment have been tried in these cases, like steroids, hyaluronic acid, lycopene, curcumin, turmeric, aloe vera, pentoxifylline, iron supplements, fibrinolytic agents, fibrotomy, and removal of fibrous bands using light amplification by stimulated emission of radiation (LASER), with varying degrees of success [3,4]. In the early stages of OSMF, conservative management like cessation of habits and medical management like antioxidants, nutritional, iron, and vitamin supplements, along with corticosteroids, are considered [1]. Treatment with intra-lesional corticosteroids, placentrex, hyaluronidase, and pentoxifylline is considered in a moderate stage, like grade III. In advanced stages where mouth opening is not adequate, surgical management with the release of fibrous bands is done, and reconstruction of the defect has been done with a buccal pad of fat, a nasolabial flap, skin grafting, and a radial forearm flap in a few studies [5]. However, to date, it has been a recurrent problem, irrespective of the type of treatment used.

Intra-lesional placental extract has been used in atrophic rhinitis by inducing sterile inflammation in the mucosa and increasing blood circulation and tissue vascularity [6]. Few authors have tried intra-lesional purified placental extract and topical application of placental extract gel following fibrotomy in oral sub-mucous fibrosis with varying degrees of success [7-10]. The objectives of our study are to document the clinical outcome of intra-lesional injection of purified human placental extract and topical application of placental extract gel after fibrotomy and compare the outcomes with regard to mouth opening, colour of the mucosa, and burning sensation in OSMF.

## Materials and methods

The study was approved by the institutional ethics committee (SDUMC/KLR/IEC/649/2020-21). This randomised controlled study was conducted in the Department of Otorhinolaryngology and Head and Neck Surgery in a rural tertiary care hospital from January 2021 to August 2022. This study included 58 patients diagnosed with oral submucous fibrosis based on an accurate clinical examination by a senior head and neck surgeon and a biopsy from the representative area with OSMF for confirmation of the diagnosis and to rule out carcinoma in situ, if any.

The sample size was estimated based on the mean difference in mouth opening pre-operatively and post-operatively as reported in the study by Thakur et al. [4]. The study reported an average variance estimate of 2.7 in the mouth opening score with 80% power and an alpha error of 5%. To detect a 14% difference, the mean score sample size was estimated per group at 29.

Informed written consent was obtained from all the study subjects to be part of the study and to undergo any of the two interventions in the study after explaining the likely outcomes and potential complications. These 58 patients had willingly given consent and qualified for the study based on the following inclusion and exclusion criteria.

Inclusion criteria

Patients with OSMF confirmed by histological examination of punch biopsy and aged between 20 and 60 years, having grade II (inter-incisor distance 25-35 mm on maximum mouth opening) or grade III (inter-incisor distance 15-25 mm on maximum mouth opening) trismus, involving the retro-molar trigone (RMT) and extending into buccal mucosa and palate with/without the involvement of the anterior pillar of the tonsil, having burning sensation in the oral cavity, particularly while consuming food, and addicted to tobacco quid containing betel leaf, tobacco, areca nut, and slaked lime. The exclusion criteria included patients with grade I (inter-incisor distance 35-40 mm on maximum mouth opening) and grade IV (inter-incisor distance <15 mm on maximum mouth opening) trismus, a history of major surgeries in the oral cavity, who had undergone treatment for OSMF, patients with syndromic disease-causing oral ulcers, patients who got treated by radiotherapy to the oral cavity in the past, and patients allergic to placental extract, oral carcinoma, or carcinoma in situ. Routine blood investigations like the complete blood count, coagulation profile, serum electrolytes, human immunodeficiency virus (HIV), and HbsAg were assessed before the procedure.

The patients were randomly allocated into two groups of 29 patients each by four block randomisation using computer software developed by Urbaniak and Plous, Research Randomizer (version 4.0), as per the online plan [11]. Both group I and group II patients were age-matched into groups spanning 10 years each within a maximum margin of five years. This way, age as a confounder was nullified.

**Figure 1:**
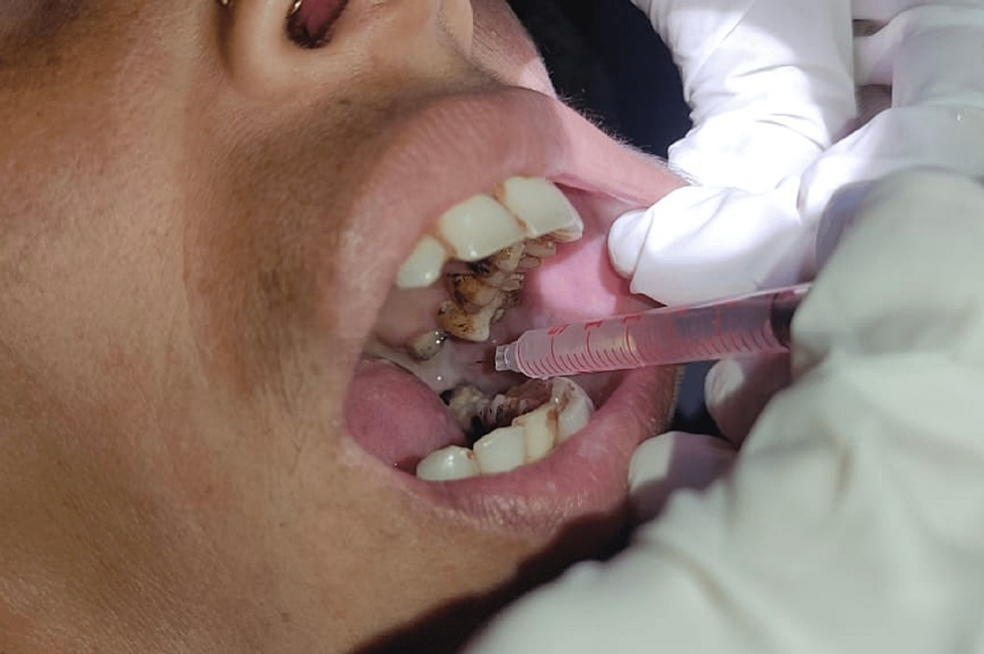
Intra-lesional placental extract injection

The maximum mouth opening was measured as the inter-incisor distance in millimetres (between the upper and lower incisors) using slide callipers, colour of the mucosa and burning sensation in the oral cavity according to the Likert scale were documented prior to treatment. Group I patients received 1 ml of intra-lesional human placental extract injection in the submucosal plane in the retro-molar trigone (where the fibrosis was maximum) and adjoining buccal mucosa laterally and medially until the anterior pillar of the tonsil once a week for five consecutive weeks (Figure 1).

**Figure 2:**
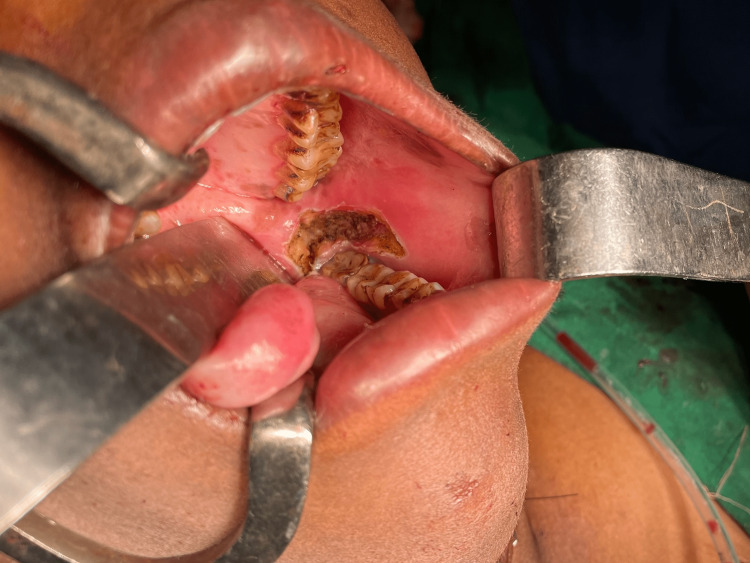
Fibrotomy done in grade II trismus

Group II patients were subjected to the transverse division of fibrotic bands in the submucosal plane from the lateral aspect of the anterior pillar of the tonsil across the retro-molar trigone and, in cases where it was extending to the buccal mucosa, up to 1 cm into the buccal mucosa (all in the submucosal plane) under general anaesthesia (Figure 2).

The surgical wound was left open, and swabs soaked in human-purified placental extract gel were placed in the wound for two hours twice daily until the surgical wound was epithelialized and healed. Povidone-iodine mouthwashes were given frequently to both groups until the surgical wound in group II healed to prevent infection. The patients in both groups I and II were advised to perform jaw-opening exercises using a Heister jaw opener every four hours at home. Weekly follow-up was done in all patients, and findings with regard to maximum mouth opening (inter-incisor distance), colour of the mucosa, and burning sensation in the oral cavity based on the Likert scale were documented. Patients were followed up for five months, during which they were asked to do regular mouth-opening exercises and maintain oral hygiene. Any improvement in mouth opening, the colour of the mucosa, and the burning sensation were documented. Any adverse drug reactions or complications after the procedure were documented. A comparison was made between the above two groups of patients five months after completing treatment. The tools for comparison between the two groups were improvement in mouth opening (inter-incisor distance measured using slide callipers in millimetres), improvement in the colour of the mucosa, and subjective relief from burning sensation in the oral cavity. At the end of five months, the patients were advised to fill out a questionnaire on a Likert scale regarding the severity of the burning sensation in the oral cavity compared with the pre-treatment period.

Findings were documented in Statistical Package for Social Sciences (SPSS) 22 version software (IBM Corp., Armonk, NY), and analysis was done. An independent t-test was used as a test of significance to identify the mean difference between two quantitative variables. A P-value (probability that the result is true) of <0.05 was considered statistically significant after assuming all the rules of statistical tests.

## Results

The total number of patients included in the study was 58, and they were divided into two groups of 29 patients each, out of which 40 were females and 18 were males. The mean age in group I patients was 48.14 (age range of 28 to 59 years), and in group II, it was 47.51 (age range of 28 to 58 years). Group 1 consisted of 17 patients (58.6%) having grade II trismus and 12 patients (41.4%) having grade III trismus. Group II consisted of 15 patients (51.3%) having grade II trismus and 14 patients (48.3%) having grade III trismus. All patients were addicted to chewing areca nuts with tobacco. Bilateral involvement was present in all of the patients, with extension into the retromolar trigone (RMT) and extension into the soft palate seen in 31%.

**Figure 3:**
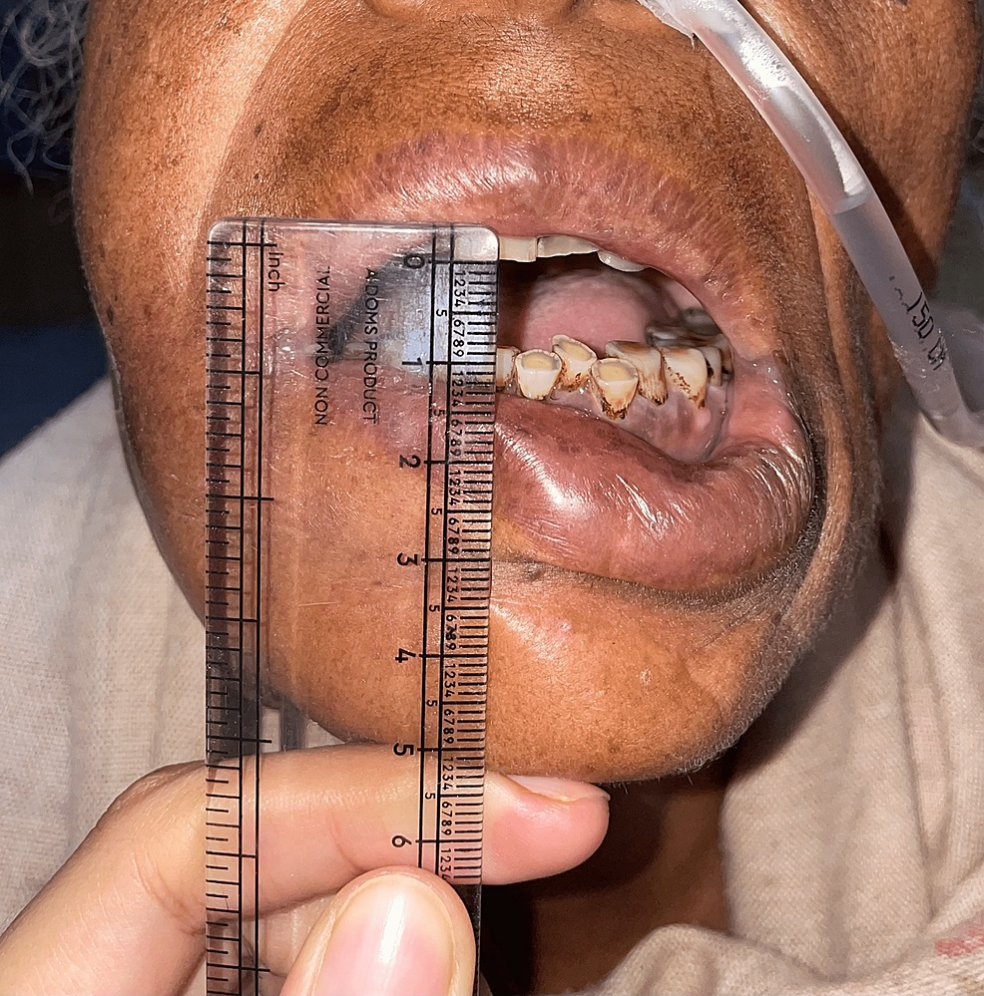
Pre-treatment mouth opening in grade III trismus

**Figure 4:**
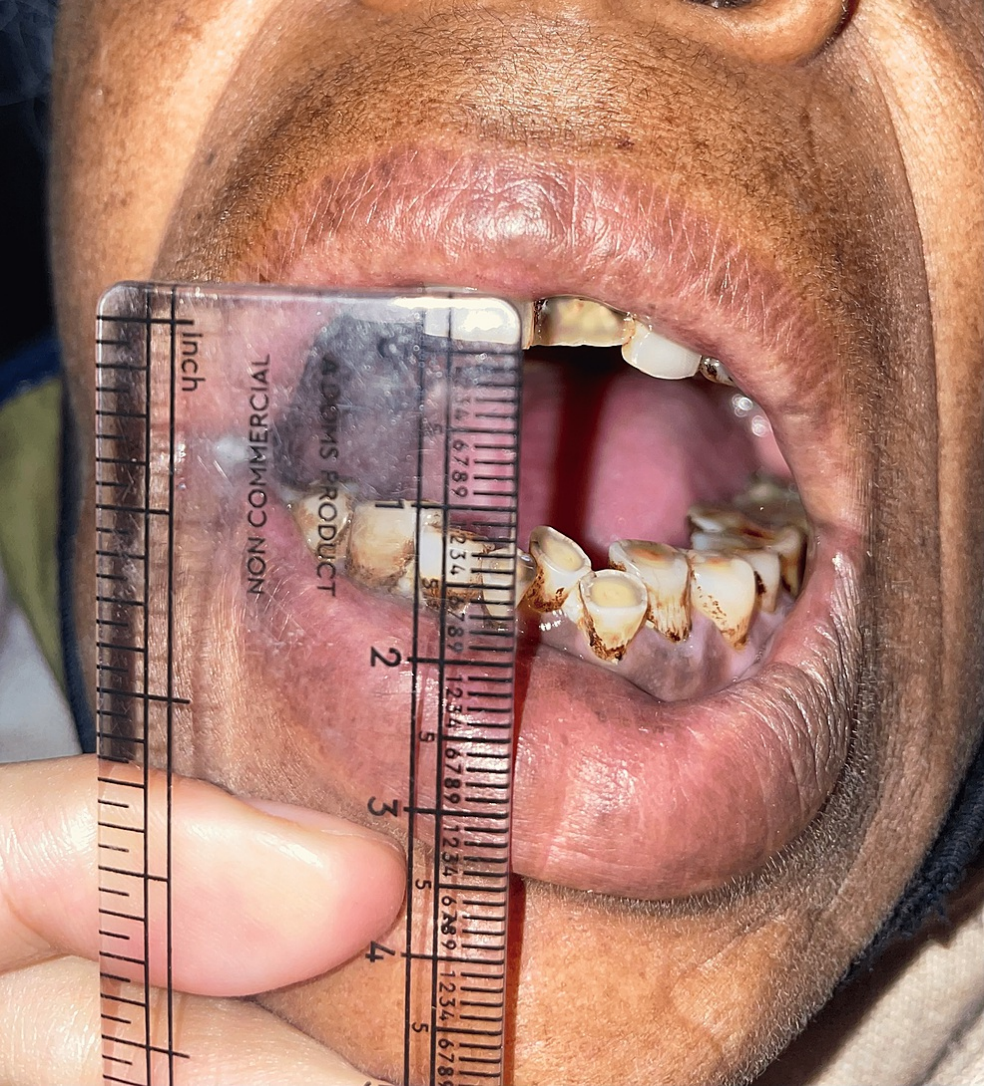
Post-treatment (fibrotomy) mouth opening after five months

Baseline mouth opening pre-treatment was 22.45 mm in group I and 21.41 mm in group II. Post-treatment mouth opening measured at five weeks from baseline was 25.21 mm in group I and 26.41 mm in group II. The mean increase in mouth opening after five weeks of treatment was 3.14 mm in group I, while it was 5.00 mm in group II, with a p-value of <0.001, which was found to be statistically significant (Figures 3-4).

Burning sensation pre-treatment in group I was mild in 13 patients (44.8%), moderate in 12 patients (41.4%), severe in one patient (3.4%), and absent in three patients (10.3%). In group II, the pre-treatment burning sensation was mild in 13 patients (44.8%), moderate in 12 patients (41.4%), severe in three patients (10.3%), and absent in one patient (3.4%). After five months, relief from the burning sensation in group I improved to mild in 14 patients (48.3%), moderate in two patients (6.9%), and absent in 13 patients (44.8%). In group II, after five months, the burning sensation was mild in 17 patients (58.6%), moderate in three patients (10.3%), and absent in nine patients (31%). No statistically significant difference (p-value 0.581) was found between the two groups with respect to the treatment outcome for burning sensation.

The colour of the mucosa before receiving treatment in group I was pale in 89.7% of patients, which improved to 72.4% of patients after five months. 89.7% of patients in group II had pale mucosa, which improved in 69% of patients after five months. No statistical difference was found between the two groups with respect to the colour of the mucosa (p-value 1.00). The mucosa looked relatively better and pinkish in group I.

The summary of results documented in the study is depicted in Table 1.

**Table 1:** Summary of results

	Group I		Group II		P-value
	Mean	SD	Mean	SD	
Comparison of mouth opening (in mm) between two groups					
Pre-treatment	22.45	4.298	21.41	4.822	0.390
After five months	25.21	4.894	26.41	4.866	0.350
Mean difference	3.14	1.529	5.00	0.886	<0.001*
	Group I		Group II		P-value
	N	%	N	%	
Distribution of subjects according to a burning sensation among the two groups					
Pre-treatment					
Absent	3	10.3%	1	3.4%	
Mild	13	44.8%	13	44.8%	
Moderate	12	41.4%	12	41.4%	
Severe	1	3.4%	3	10.3%	
After five months					
Absent	13	44.8%	9	31.0%	
Mild	14	48.3%	17	58.6%	
Moderate	2	6.9%	3	10.3%	
Distribution of subjects according to the colour of mucosa among the group					
Pre-treatment					
Pale	26	89.7%	26	89.7%	
Pinkish	3	10.3%	3	10.3%	
After five months					
Pale	21	72.4%	20	69.0%	
Pinkish	8	27.6%	9	31.0%	

## Discussion

OSMF is a chronic, insidious, pre-malignant condition of the oral cavity characterised by inflammation and proliferation of fibroblasts in the lamina propria of the mucosal layer. It is a debilitating condition that gradually reduces the mouth opening, causes ulcers and a burning sensation in the mouth, makes it difficult to eat and swallow, causes nutritional deficiencies and bad oral hygiene, and affects one's ability to communicate [12]. Areca nut chewing is the main risk factor causing OSMF. The constituents of the areca nut and its metabolites also irritate the oral mucosa. Arecoline from Areca catechu is the main causative factor in addition to pan and tobacco in different combinations [13]. They are also rich in copper, which causes constant chewing of areca nuts to increase the amount of copper content in the oral cavity fluid. Copper is also one of the factors that cause stimulation of fibroblasts, leading to fibrogenesis, by up-regulating the activity of copper-dependent lysyl oxidase [14].

According to Pindborg et al., the prevalence rate was up to 0.4% in the Indian rural population [15]. A study conducted by Pandya et al. states that India has a prevalence of 0.2-0.5% [16]. OSMF has a malignant transformation rate of 9.13% and a 29.26 times higher risk of developing cancer than those who do not [17].

Our study included 58 patients. Among them, 40 were female and 18 were male. The mean age in group I patients was 48.14 (age range 28 to 59 years), and in group II, it was 47.51 (age range 28 to 58 years). In rural areas, people get addicted to chewing tobacco at a young age, which explains the wide age range among both groups. This is in contrast to Naik et al. and Reddy et al., who observed a male preponderance [8,9]. The reason for the high prevalence of oral cancer among females in our region can be explained by females' addiction to tobacco quid, which is sometimes kept overnight. All patients have the habit of chewing areca nuts with tobacco. Bilateral involvement was present in all of the patients, with extension into the RMT and soft palate extension seen in 31%.

Naik et al. compared intra-lesional triamcinolone acetonide and hyaluronidase with placental extract and observed that the combination of triamcinolone acetonide and hyaluronidase showed a better outcome than placental extract injection with an increased inter-incisor distance after eight weeks from 16 mm to 35 mm in the group receiving a combination of triamcinolone acetonide and hyaluronidase and from 15 mm to 33 mm in the group receiving intra-lesional placental extract injection [8]. In our study, the mean increase in mouth opening in group I after five months was 3.14 (3-5 mm) and in group II was 5.00 (4-6 mm) with a p-value of <0.001, which was statistically significant. Our outcomes were similar to those of a study by Shah et al., where they compared a combination of the placental extract with dexamethasone injection and hyaluronidase with dexamethasone injection. The mean increase in mouth opening after eight weeks in their study was between 3.5 and 3.6 mm [10]. The limited improvement in mouth opening in both groups in our study may be because the patients were advised jaw opening exercises at home without an occupational therapist, which might have led to poor compliance, and the release of fibrous bands was done in the retro-molar trigone and adjoining few millimetres. Extension of the fibrotomy along the length of the buccal mucosa may show better improvement in mouth opening. In our study, no healthy vascularised tissue was interposed in the fibrotomy wound. The addictions to carcinogens in our study were similar to those in other studies and literature.

Kisave et al. compared the injection of placental extract and hydrocortisone and concluded that better mouth opening was achieved by hydrocortisone injections [12]. However, a study conducted by Reddy et al. compared the efficacy of corticosteroids and placental extract injections. Patients who received submucosal injections of placental extract showed better outcomes, though the results were not statistically significant [9]. Our study group, which received intralesional placental extract injections, also showed similar outcomes.

Chaudhry et al. performed fibrotomy using LASER in 16 cases with moderate OSMF under local anaesthesia and found that the mean increase in mouth opening after a period of one year was 17.5 mm [13]. Thakur et al. performed a fibrotomy followed by applying placental extract gel topically over the wound in 10 patients where the mouth opening was less than 20 mm and a buccal pad of fat was used for reconstruction. They found that the median postoperative improvement in mouth opening after four weeks was 15 mm [4]. The mouth opening in the above-mentioned series was much better than the outcome in our study. A longer follow-up with jaw-opening exercises under supervision might have given a better outcome in this study.

The colour of the mucosa before receiving treatment in group I was pale in 89.7% of patients and improved in 72.4% of patients after five months. 89.7% in group II had pale mucosa, which improved to 69% after five months. No statistical difference was found between the two groups with respect to the colour of the mucosa, which was similar to the outcomes of Naik et al. [8].

Burning sensation pre-treatment in group I was mild in 13 patients (44.8%), moderate in 12 patients (41.4%), severe in one patient (3.4%), and absent in three patients (10.3%). In group II, the pre-treatment burning sensation was mild in 13 patients (44.8%), moderate in 12 patients (41.4%), severe in three patients (10.3%), and absent in one patient (3.4%). After five months, relief of the burning sensation in group I improved to mild in 14 patients (48.3%), moderate in two patients (6.9%), and absent in 13 patients (44.8%). In group II, after five months, the burning sensation was mild in 17 patients (58.6%), moderate in three patients (10.3%), and absent in nine patients (31%), which led to the conclusion that there was better relief of the burning sensation among the group I patients who received intra-lesional placental extract injection. However, it was not statistically significant between the two groups in our study.

Chaudhary et al. compared the pre- and post-operative burning sensation using a visual analogue score and found it to be statistically significant [13]. Naik et al. also concluded that both groups of patients showed equal improvement with relief of the burning sensation and colour of the mucosa [8]. Kisave found that patients receiving placental extract injections had better relief of burning sensations after 12 weeks with hydrocortisone compared to placental extract injection [12].

In our study, all of the patients were clinically diagnosed with OSMF, with the majority of them having an associated burning sensation. The results of the group that underwent fibrotomy with topical application of placental extract gel showed a statistically significant outcome. Although the outcome of the burning sensation and colour of the mucosa was not statistically significant, all patients had relief of symptoms and improvement in the colour of the mucosa. As the mouth-opening exercises and application of placental extract gel were not always supervised, larger multi-institutional studies with occupational therapists may contribute to a better outcome.

Limitations

Limitations of this study include the small sample size and the short duration of follow-up. Also, patients were not monitored, so they may not do regular mouth-opening exercises at home. The exact protocol for interventional studies is difficult to follow in a developing country, especially in rural areas where the population is largely uneducated and economically poor and depends on the daily wage for their income.

## Conclusions

The fibrotomy of submucous fibrous bands followed by topical application of human placental extract gel gives better results with regard to mouth opening in OSMF. Symptomatic relief in burning sensation and change in colour of mucosa was better among the patients who received intralesional placental extract injection. However, the difference was not statistically significant. Extension of fibrotomy along the length of the buccal mucosa with aggressive jaw opening exercises and larger multi-institutional studies with the involvement of occupational therapists may be required for more validated outcomes.
